# Clinical Reports

**Published:** 1860-07

**Authors:** N. S. Davis

**Affiliations:** Mercy Hospital


### CLINICAL REPORTS.

Mercy Hospital, June 21st, 1860. Service of Dr. N. S. Davis.

The substance of the clinique this morning was as follows:

Mr. M., a native of Ireland, aged about thirty years, was admitted into the Hospital between three and four weeks since. He was thin in flesh, with a depressed and anxious expression of countenance; pulse frequent, but not full; skin hot; tongue coated with a thick white fur; considerable thirst; bowels slightly relaxed; and a constant dorsal position. He kept the left thigh flexed upon the pelvis; complained of a very severe paroxysmal pain in the outer part of the left groin, extending at times down the anterior part of the thigh, which was greatly increased by every attempt to move the limb. The groin was tender to pressure, but not visibly swollen. Alteratives and anodynes were given internally, and cloths wet in the infusion of Aconite Leaves applied to the groin and upper part of the thigh. In three or four days the general febrile disturbance ceased, and the pain changed from the groin to the hip or gluteal region, extending from the left side of sacrum to the level of trochanter major, and sometimes following the whole length of the sciatic nerve and its branches to the toes. There was a constant dull pain, with frequent paroxysms of great severity. The paroxysms were often accompanied by spasmodic action in the flexor muscles of the limb. The whole gluteal region, and especially the trunk of the sciatic nerve and its origins from the spine, was tender to the touch. The thigh was partially flexed upon the pelvis, and the knee turned in, resting against the knee of the opposite side, and every attempt to move it from that position caused the most excruciating pain. The skin was relaxed, and almost constantly bathed in perspiration.

The attention of the class was called to the symptoms of this case minutely, as it involved the diagnosis between psoas inflammation and abscess, hip-joint disease, and sciatica. It was noticed that the pain commencing in the groin, and the flexed position of the limb with the knee turned inwards, corresponded with the phenomena of psoas abscess, or at least irritation along the upper part of the psoas muscle. But the sudden change of the pain from the groin to the outer part of the hip, leaving in the iliac and psoas regions, neither pain, swelling, or tenderness, together with the absence of the rigors and hectic which usually mark the commencement of internal abscesses, renders it almost certain that no inflammation or suppuration exists in the iliac or psoas regions. Reviewing the symptoms of hip-disease in comparison with this case, we shall find the following marked differences. In the ordinary form of Coxalgia or hip-disease, it commences very slowly by a simple awkwardness in walking, and generally neuralgic pains referred to the knee, and many months will elapse before the patient becomes wholly disabled, thus differing entirely from the progress of the case before us. However, we sometimes meet with cases of a more acute inflammation of the sinovial membrane of the hip-joint which may develope itself rapidly, characterized by great pain, increased by every movement of the limb, and more or less general febrile symptoms. But in all such cases there is early and marked swelling, with acute tenderness directly in the region of the joint; while here there is no swelling, although the patient has been confined to the bed for several weeks, and no tenderness except in the track of the sciatic nerve. Again, in hip-disease, whether acute or chronic, pressure on the trochanter major in such direction as to press the head of the femur into the acetabulum pretty uniformly causes pain, while here no pain is occasioned by such pressure. We thus find by a close comparison that some of the essential phenomena of both psoas abscess and coxalgia are absent, while the prominent symptoms actually present are such as might result from irritation of the sciatic nerve, or of that part of the spinal cord from which it originates. Hence, it would be called a case of Sciatica. To get a rational basis for treatment, however, we must pursue the subject of diagnosis still further. For at the bed-side we have found *three* varieties of disease involving the sciatic nerve, not only differing in their pathology, but also in the therapeutic means required for their treatment. The first consists in an inflammation of the neuralema or fibrous sheath investing the nerve, and is generally of rheumatic origin. It is characterized by a dull, aching pain, extending from the lumbar vertebræ to the upper and outer part of the thigh, with irregular exacerbations of great acuteness, and extending through the whole length of the limb to the toes. There is acute tenderness over the origins and trunk of the nerve, but without swelling, and the pain is more severe at night, and greatly aggravated by any movement of the limb. In the early stage it is often accompanied by slight general fever, and sometimes by rheumatic inflammation in other parts of the body. The second variety is characterized by severe paroxysms of pain in the nerve, generally commencing behind and above the trochanter, and extending more or less down the limb, but strictly periodical in their occurrence; that is, the paroxysms commence about the same time every day or every second day, continue a given number of hours and then cease, with as much regularity as the paroxysms of an intermittent.

There is usually no fever, and if slight tenderness exists during the paroxysms, it entirely disappears in the intermissions. This variety is undoubtedly of malarious origin, being chiefly met with in districts where intermittents prevail endemically, and may be properly styled periodical sciatica.

The third variety is characterized by irregularly recurring paroxysms of very acute pain in the course of the nerve, commencing as suddenly as a current of electricity, and ceasing equally sudden, and unaccompanied by either fever or tenderness to pressure. When it has continued for several months, the muscles of the limb are generally found to be more or less atrophied, and their contractility so much impaired as to produce a clumsy or awkward gait in walking.

Your attention is called thus minutely to the several varieties of Sciatica; that you may avoid the very common practice of applying certain remedies empirically to all cases, without any reference to their special characteristics.

Both the causes and the pathology of the third variety named are involved in obscurity and doubt; and the majority of cases seem to be but little influenced by remedial agents. The second class of cases more generally yield readily to a judicious use of anti-periodics and tonics. The first class of cases, to which the patient before us evidently belongs, are generally amenable to such remedies as relieve sub-acute rhuematism in other parts of the body. If we have correctly interpreted the symptoms of this case, it consists of a sub-acute rheumatic inflammation of the neuralema or sheath of the sciatic nerve from its origin in the spinal cord to a point a little below the level of the trochanter major. We have before explained that all inflammations when closely analized are found to contain three elementary pathological conditions, namely, an exalted susceptibility in the structure, an altered affinity, and an accumulation of blood in the capillaries; and that these several conditions exist in very variable degrees of intensity in different cases, thereby causing the different varieties of inflammation. In all rheumatic inflammations, the first element, which we call exalted susceptibility, predominates, causing great pain and sensibility of parts, with comparitively little change either in the accumulation of blood or the nutrition of the part. This is pre-eminently true when, as in the present case, the inflammation is in parts immediately investing nerve matter. Hence, in examining this man's hip, you do not find sufficient accumulation of blood in any of the structures to cause a perceptible degree of swelling, or even increased local temperature, and yet the sensibility is so much exalted that every motion or touch causes the most severe pain, while the spasmodic action of the muscles show the same exaggerated influence of the motor filaments of the nerves.

These conditions have an important bearing on our therapeutic measures. If the inflammation was located in a highly vascular structure like the lungs, for example, the accumulation of blood might be so great, that with the altered vital affinity, a dangerous degree of engorgement and infiltration of texture would result. Hence, measures calculated to counteract or relieve such accumulation of blood would constitute a primary indication in the treatment. But in the case before us, the small extent of the texture involved, and the little comparitive vascularity, renders this indication of minor importance, while the extreme exaltation of susceptibility in the enclosed nerve structure calls for the use of such agents as tend to subdue this as a primary step in the treatment. You are already aware, from previous instruction in these wards, that rheumatic inflammation is very generally regarded as arising from the retention of some effete and disturbing element in the blood; and consequently that the first object of the physician should be, either to expel by elimination, or neutralize by chemical agents, this supposed irritant.

Without denying the correctness of this doctrine in relation to the essential cause of rheumatic inflammation, we must caution you against a prevalent tendency to restrict our attention entirely to this and the remedial measures it suggests. You must remember that an inflammation, or any other morbid process, does not always cease with the ceaseation of the efficient cause which excited it to action.

Hence, though a removal of the cause may be a primary indication, it does not necessarily supersede all other indications for treatment. On the contrary, a morbid process, like inflammation, once established in any given structure may persist, accompanied by great pain, until it results in serious change of texture by effusion, infiltration, and induration; or in destruction by suppuration and gangrene.

For reasons already given, we have little to fear from any of these changes of texture in the case before us; consequently it presents but two prominent objects to be accomplished by treatment. First, to remove the exciting cause; and second, to overcome the extreme susceptibility and consequent pain which has been engendered in the affected nerve and the parts on which it is distributed. On the first admission of the patient, we endeavored to accomplish these two objects by using such remedies as promote excretion in conjunction with such as directly diminish morbid susceptibility.

We gave the following:

| | | |
|---|---|---|
| ℞ | Vin. Colchici | ℥ j. |
| | Tinct. Cimicifuga Rac. | ℥ ii. |
| | Tinct. Verat. Viride, | ʒ i. |

Mixed, of which a teaspoonful was given every four hours, with the following powder at bed-time:

| | | |
|---|---|---|
| ℞ | Pulv. Opii, | 2 grs. |
| | Nit. Potassa, | 10 grs. |
| | Hydrarg. Chlorid. Mite. | 2 grs. Mix, one powder. |

At the same time we kept the whole groin and hip covered with cloths wet in a warm infusion of Aconite Leaves. In less than forty-eight hours the general febrile symptoms had disappeared, the skin and kidneys acted freely, the tenderness of the nerve tracks was diminished, but the paroxysms of pain and spasmodic action of the muscles of the thigh continued. The Tinct. Verat. Viride was now omitted from the first prescription, and otherwise the same treatment continued. The next day the pain and tenderness had left the groin entirely, but remained severe in the origins and trunk of the sciatic nerve. The patient had sweat copiously, and the kidneys had acted freely during the whole of the past three days, and now the Colchicum began to move the bowels too freely. We continued the same applications externally, and gave internally the following;

℞ Pulv. Opii, 10 grs.
Nit. Potassa, 30 grs.
Pulv. Doveri, 30 grs.

Mix, and divide into six powders, of which give one every four hours.

Having occasion to leave home for ten days, all my patients here were left in charge of my colleague, Prof. Andrews.

You will perceive that up to this time the treatment had been designed, on the one hand, to promote elimination sufficient to free the system from any retained effete or disturbing agents, and on the other, to allay pain and morbid sensibility. As the relief of the patient was only partial, my colleague adopted a course of treatment more directly calculated to act chemically upon the fluids of the body, by giving the patient freely of the Salts of Soda and Potassa, with an anodyne at bed-time. Still, as you have just seen by a careful examination of the patient, it has exerted very little apparent influence over his suffering.

The questions now arise, why does the pain continue? And what further remedial agents are called for? You have already been cautioned against relying too exclusively upon such means as are calculated merely to remove the exciting cause of a disease. The case directly before us proves the necessity for that caution. For three weeks all the important excretory organs have been kept active, and for more than one week the fluids of the body have been freely saturated with alkaline salts. Hence, if the disease depended upon the presence of any acid irritant in the system, as the prevailing doctrines of the profession claim, it certainly should have been either eliminated or neutralized. It is true that under the past treatment all general fever has disappeared, and the local tenderness has much diminished, but the pain and spasms, depending, as we suppose they do, on the irritation or morbid susceptibility of the nerve structure, still continue. May not this continuance of nerve irritation depend, in part at least, on too great a relaxation of structures, or in other words, debility, favored by the excessive eliminations of the past two or three weeks?

Whatever may be the theories we adopt for explaining the condition of the patient, it is certain that he is now much debilitated, and all his structures relaxed, and yet a fixed position of the limb, with frequent and excessive paroxysms of pain. Hence, the indication for further treatment plainly consists in the use of such agents as will strongly diminish nerve irritability and pain, while they increase the vital affinity and consequent tonicity of the tissues. For this purpose no more efficient combination can be found than that of Opium with Quinine, given in moderately large doses. Sometimes, however, Opium when thus administered for several days in succession, checks too much the action of the kidneys. This can be prevented by adding Nitrate of Potassa to the other ingredients. We shall therefore direct for this patient the following:

| | | |
|---|---|---|
| ℞ | Sulph. Quinine, | 24 grs. |
| | Pulv. Opium, | 16 grs. |
| | Nit. Potassa, | 40 grs. |

Mix, and divide into eight powders. Give one every four hours.

At the same time we shall cause a small blister to be made just above the left sacro-iliac junction, the cuticle to be removed, and half a grain of Morphia applied to it each night and morning, with a dressing of mild Mercurial Ointment during the interval. If the pain becomes subdued and the patient sleeps, we shall gradually diminish the quantity of both quinine and opium, aiming to so adjust the doses as to keep the pain under control without stupifying the patient. If necessary, a fresh blister will be made over the track of the sciatic nerve every two or three days.

*Note.*—Two weeks have now elapsed, and the patient is slowly recovering without any material alteration of the plan of treatment just set forth.

